# Use and impact of the prehospital 12-lead ECG in the primary PCI era (PHECG2): protocol for a mixed-method study

**DOI:** 10.1136/openhrt-2019-001156

**Published:** 2019-12-02

**Authors:** Lucia Gavalova, Mary Halter, Helen Snooks, Chris P Gale, Clive Weston, Alan Watkins, Scott Munro, Glenn Davies, Chelsey Hampton, Timothy Driscoll, Andy Rosser, Nigel Rees, Sarah Black, Tom Quinn

**Affiliations:** 1 Emergency, Cardiovascular and Critical Care Research Group, Kingston University and St. George's, University of London, London, UK; 2 Medical School, Swansea University, Swansea, West Glamorgan, UK; 3 Leeds Institute for Cardiovascular and Metabolic Medicine, University of Leeds, Leeds, UK; 4 Glangwili General Hospital, Carmarthen, Carmarthenshire, UK; 5 South East Coast Ambulance Service NHS Foundation Trust, Crawley, UK; 6 Patient and Public Representative, London, UK; 7 West Midlands Ambulance Service NHS Foundation Trust, Brierley Hill, West Midlands, UK; 8 Research and Development Office, Welsh Ambulance Services NHS Trust, Saint Asaph, Denbighshire, UK; 9 Research, Audit and Improvement, South Western Ambulance Service NHS Foundation Trust, Exeter, UK

**Keywords:** 12 lead ECG, acute coronary syndrome, quality of care and outcomes, emergency medicine

## Abstract

**Introduction:**

Use of the prehospital 12-lead ECG (PHECG) is recommended in patients presenting to emergency medical services (EMS) with suspected acute coronary syndrome (ACS). Prior research found that although PHECG use was associated with improved 30-day survival, a third of patients (typically women, the elderly and those with comorbidities) under EMS care did not receive a PHECG.

The overall aim of the PHECG2 study is to update evidence on care and outcomes for patients eligible for PHECG, specifically addressing the following research questions: (1) Is there a difference in 30-day mortality, and in reperfusion rate, between those who do and those who do not receive PHECG? (2) Has the proportion of eligible patients who receive PHECG changed since the introduction of primary percutaneous coronary intervention networks? (3) Are patients that receive PHECG different from those that do not in terms of social and demographic factors, or prehospital clinical presentation? (4) What factors influence EMS clinicians’ decisions to perform PHECG?

**Methods and analysis:**

This is an explanatory, mixed-method study comprising four work packages (WPs). WP1 is a population-based, linked-data analysis of a national ACS registry (Myocardial Ischaemia National Audit Project). WP2 is a retrospective chart review of patient records from three large regional EMS. WP3 comprises focus groups of EMS personnel. WP4 will synthesise findings from WP1–3 to inform the development of an intervention to increase PHECG uptake.

**Ethics and dissemination:**

The study has been approved by the London-Hampstead Research Ethics Committee (ref: 18LO1679). Findings will be disseminated through feedback to participating EMS, conference presentations and publication in peer-reviewed journals.

**Trial registration number:**

NCT03699137

**Key questions**

- The prehospital 12-lead ECG (PHECG) is associated with better survival in patients with acute coronary syndrome (ACS), but is potentially underused.
- In this study, we will examine whether the proportion of patients who receive PHECG has changed in the primary percutaneous coronary intervention era. In addition, we will look at barriers and facilitators of PHECG use in terms of patient-related factors and emergency medical services personnel perspectives.
- Findings from this study will provide an insight into PHECG use in patients with ACS and allow the development and testing, in a randomised trial, of an intervention to improve uptake.

## Introduction

The performance of a 12-lead ECG is recommended in the assessment of patients with suspected acute coronary syndrome (ACS) presenting in the community to emergency medical services (EMS).^1–3^ A prehospital 12-lead ECG (PHECG) informs decision-making in three components of immediate care: targeted prehospital treatment, transport to an appropriate receiving hospital and provision of information required to activate a response from the receiving cardiac catheter laboratory when ST-segment elevation myocardial infarction (STEMI) is suspected.^4^

This study builds on our previous research, which reported an association between PHECG and lower short-term mortality following STEMI and non-STEMI (30-day mortality 7.4% vs 8.2%, OR 0.94, 95% CI 0.91 to 0.96).^5^ Earlier research focused on the association of PHECG with process-of-care quality descriptors such as ‘call-to-reperfusion’ time.^6 7^ A subsequent systematic review and meta-analysis has confirmed the association of PHECG with improved clinical outcomes,^8^ and this evidence has been incorporated into international guidelines and quality indicators for prehospital care of patients with ACS.^2 3^ However, we found PHECG was underused, particularly in older patients, women and people with comorbidities.^5^

We showed that women were less likely to receive PHECG than men (OR 0.87, 95% CI 0.86 to 0.89),^5^ and suggested that this may be because the majority of EMS personnel at that time were male. The lower rate of PHECG in women was also reported from the national SWEDEHEART (The Swedish Web-system for Enhancement and Development of Evidence-based care in Heart disease Evaluated According to Recommended Therapies) registry (OR 0.89, 95% CI 0.87 to 0.92),^9^ though these authors offered a different explanation related to ‘uncontrolled confounding of presenting symptoms and type of myocardial infarction’. A systematic review reported that women are less likely to present with chest pain and more likely to have prior heart failure, which may ‘mask’ the true diagnosis.^10^

Emergency situations force clinicians into a reliance on past experiences,^11 12^ intuition and personal decision rules (‘rules of thumb’),^13^ even though these approaches are potential sources of error and bias.^14^ In prehospital emergency care, suggested influences on clinical decisions include a desire to ‘cover one’s back’,^15^ pragmatism (busy shift or end of a shift)^16^ and multilevel system influences.^17^

Since our earlier study there has been significant change in reperfusion strategies for STEMI in the UK, with primary percutaneous coronary intervention (pPCI) superseding fibrinolytic treatment as the treatment of choice.^18^ This has required changes in EMS organisation and practice, including direct transportation to pPCI-capable hospitals and the atrophy of existing programmes of prehospital administration of thrombolytic treatment by ambulance paramedics.

Considering this major change in practice and an increased emphasis on PHECG in guidelines, we expected there to be an increase in the use of PHECG since our first report. On the contrary, it appears to have declined to 64% for STEMI and 44% for non-STEMI, respectively, in 2015–2016 (National Institute for Cardiovascular Outcomes Research (NICOR), personal communication). It is therefore timely to update our analysis on a more recent cohort of patients and to explore potential determinants of why PHECG is/is not recorded.

Our a priori hypotheses are:

- PHECG use by EMS personnel is associated with more timely processes of care and a lower short-term mortality rate in patients with suspected ACS, for both STEMI and non-STEMI cases.
- Patients who do not receive a PHECG systematically differ from those who do.
- EMS clinician decision-making regarding PHECG is influenced by more than clinical (pathophysiological) characteristics.
- It is possible to use the findings from this study as the first stage of the development and subsequent evaluation of a complex intervention^19^ designed to improve the uptake of PHECG.

Our research questions (with their respective work packages (WP)) are:

- For patients presenting to EMS with suspected ACS, is there a difference in 30-day mortality and reperfusion strategy in patients managed outside hospital by EMS between those who do and those who do not receive a PHECG? (WP1)
- Has the proportion of eligible patients who received a PHECG changed since the national rollout of pPCI networks? (WP1)
- Are patients that receive a PHECG different from those that do not in terms of social and demographic factors, and in their prehospital clinical presentation? (WP2)
- What factors do EMS clinicians report as influencing their decision to perform a PHECG? (WP3)

## Methods and analysis

To answer our research questions an explanatory, mixed-method study will be undertaken,^20^ in which the qualitative data will be used to aid understanding of the quantitative findings, consisting of the following four WPs:

*WP1—population-based, linked cohort study using Myocardial Ischaemia National Audit Project (MINAP) data from 2010 to 2017* to update evidence on care and outcomes for patients eligible for PHECG. MINAP is a comprehensive registry of ACS hospitalisations mandated by the Department of Health. Each MINAP entry provides patient demographic and clinical details of the patient journey across 122 data items. Data collection and management have been described previously.^21^

*WP2—retrospective chart review*^22^*of EMS records* to collect data on factors recorded by EMS staff that may be associated with PHECG use, but are not routinely collected in MINAP.

WP3—EMS clinician self-report on PHECG recording, using focus groups.

*WP4—synthesis of the findings from WP1, WP2 and WP3*. This will be conducted to address our research questions, within our aim of using qualitative findings as explanatory of quantitative results^20^ and with the understanding that these data are intended as the development phase for the design of a complex intervention for further testing.^19^

### Study setting

WP1 will use existing data from MINAP, covering England, Wales and Northern Ireland.

For WP2 and WP3, data will be collected at three participating EMS in England and Wales.

### Study population

WP1: patients will be eligible for the study if aged 18 years or older when attended by EMS and admitted with ACS between 1 January 2010 and 31 December 2017 to one of 228 participating hospitals in England, Wales and Northern Ireland mandated to enter data into MINAP. For patients with multiple admissions, only the earliest record of their ACS event in MINAP will be used.

MINAP is overseen by a multiprofessional Domain Expert Group of stakeholders within the National Cardiac Audit Programme (NCAP). As such, this study will include data collected on behalf of the British Cardiovascular Society under the auspices of NCAP, in which patient identity is protected.^21^

WP2 will include a stratified random sample of patients with suspected ACS (by initial diagnosis in MINAP) attended by one of the three participating EMS during the study period. It will include those with or without PHECG.

WP3 will include a purposive sample of EMS clinicians of different clinical grades, with varying levels of experience in caring for patients with ACS in the prehospital setting, who are trained in the use of PHECGs and employed by one of the three participating EMS.

### Main outcome measures

WP1—all-cause 30-day mortality (the proportion of patients who die from any cause within 30 days of the date of their event) calculated from linked MINAP and Office for National Statistics (ONS, Civil Registry) mortality data.

- Proportion of eligible patients for whom PHECG performed over time.

WP2—factors that may influence the use of PHECG in ACS (eg, patient symptoms, prehospital diagnosis, recognition of ACS, EMS clinician training level and gender, patient refusal, patient ethnicity, language spoken, capacity to communicate and/or consent).

WP3—an understanding of influences on clinical decision-making regarding recording of the PHECG in ACS (and thereby, potential explanations of quantitatively measured rates of PHECG use).

### Estimated sample size

WP1: all eligible patients in MINAP will be included in analysis. Formal power calculations are therefore unnecessary, but we anticipate that data extraction and linkage will generate a dataset comprising over 500 000 cases.

WP2: we will sample 1650 cases at random from the MINAP database using a stratified sampling method. We will oversample by 10% (165 cases) to allow for any cases where the corresponding EMS clinical records cannot be located. Based on information received from NICOR, we expect approximately a 2:1 ratio of PHECG cases to non-PHECG cases (NICOR, personal communication). Assuming this, we will have 80% power (using 5% significance) in detecting a standardised statistical effect of 0.15.

WP3: six EMS clinician focus groups (two per EMS), each comprising up to eight participants, will be convened, purposively sampling to capture the views of staff in both urban and rural settings within large EMS. We aim to obtain depth with a sample size that is neither too small to contain various aspects of the phenomenon nor too large to allow depth of analysis in this qualitative study.^23^

### Data collection

WP1: we will use existing MINAP registry data, collected prospectively at each hospital using a secure electronic system, encrypted and transferred online to a central database in which patient identity is protected.^21^ We will link MINAP data to ONS Civil Registry mortality data at individual level through NHS Digital.

WP2: we will randomly select a cohort of patients with and without PHECG, with ACS recorded in MINAP, for the three participating EMS using a unique identifier for patients in MINAP who came to hospital via EMS. The sample will be stratified by preagreed demographic variables (such as sex and age band) to ensure it is sufficiently representative. We will report key characteristics of the sample, those of the MINAP ACS subpopulation (those who came via EMS), and the overall MINAP population.

We will employ and train paramedic researchers at each site to retrieve and extract data from the EMS records of each sampled patient. We will collect data on factors that may influence the use of a PHECG which are not recorded in MINAP, including coded data on patient symptoms, prehospital diagnosis of ACS, EMS clinician training level and sex, patient refusal, patient ethnicity, language spoken, capacity to communicate and/or consent. The paramedic researchers will extract relevant narrative data from the ‘free-text’ section within EMS records. A second researcher will independently extract data for a random sample of 10% of the cases for quality assurance purposes.

WP3—we will conduct focus groups using a semistructured approach, aiming to explore the views of EMS clinicians about the role of PHECG, experiences of assessing patients with STEMI and non-STEMI, and influences on whether or not to record a PHECG. A topic guide will be constructed in the context of the quantitative findings and with input from EMS clinicians, patient and public representatives, and in the context of literature that highlights complexity and multiple influences on decisions made in the EMS setting. Focus group discussions will be digitally recorded and transcribed verbatim. Participants will be briefed at recruitment and at the beginning of each focus group not to disclose any information that might serve to identify individual(s).

### Analysis

WP1—available demographic data will be presented using interval categories for continuous variables where appropriate. We will present mortality as (1) the proportion occurring within 30 days of hospitalisation, and (2) time to death, summarised using Kaplan-Meier survival curves. We will estimate (with 95% CIs) the outcomes associated with the performance of PHECG via a binary variable, adjusting for other statistically significant demographic and clinical variables.

A similar modelling approach will be used for other categorical variables of interest (for instance, use of reperfusion); natural extensions within the framework of generalised linear models will cover both the analysis of any counts and measurement outcomes of interest.

WP2—partitioning our sample of ACS cases by PHECG or not, we will use univariate and multivariate analyses to explore differences in patient symptoms and other factors between these two groups; we will report and explore differences across stratifying variables (such as age band, sex), and across categorisations emerging from consideration of free-text narratives in EMS records). We will use binary logistic regression to explore the extent to which factors are associated with PHECG use or not in ACS cases, reporting odds ratios (with 95% CIs) for statistically significant variables. Inter-rater reliability of data retrieval will be assessed using Cohen’s kappa.

WP3—focus group transcripts will be managed using NVivo software.^24^ Initially, we will conduct an inductive thematic analysis, using bracketing, and with iteration to the coding framework based on deep reading of the data, discussion within the research team and member checking. We will then use framework analysis^23^ and analyse whether the generated themes show consensus or dissent when considered against any differences in the characteristics of the focus group participants (eg, grade of EMS clinician, local practice). At least two researchers will read, reread and draft a coding framework for discussion and blinded application to at least two transcripts. The coding framework will be developed iteratively by discussion throughout the analysis process. Researchers involved in the qualitative data collection and analysis will meet regularly to reflect on their assumptions, personal influences and data interpretations.

WP4—evidence from the WPs will be presented to a wider consultative group of study participants including local EMS collaborators and patient and public representatives. This wider group will be invited to discuss and help the research team interpret the emerging findings. WP leaders will collaborate through discussion to reach agreement on the evidence against each research question.

### Patient and public involvement (PPI)

Patients and public representatives (PPI) have been involved in the study since its inception. They played a significant role in the development of the research questions, understanding their relevance to the problem area, the study proposal and the funding application. PPI colleagues’ involvement will continue through their participation in the study oversight activities, in the interpretation of the study results and subsequently in the dissemination of the findings via their local networks and other appropriate routes.

### Clinical and public health implications

Understanding the clinical and non-clinical factors influencing EMS clinicians’ decisions to record a PHECG will enable us to develop (and later, test in a randomised trial) an intervention with the potential to improve PHECG uptake and patient outcomes following an ACS event. With over 85 000 admissions with acute myocardial infarction recorded in MINAP each year, and millions worldwide, the potential for this research to improve outcomes nationally and internationally is substantial.

### Strengths and limitations of the study

The main limitation of the study is its observational, cross-sectional nature, which prevents us from inferring causal relationships. Retrospective chart review methods, although commonly used in emergency research, are not without shortcomings^22^; for example, they are subject to bias by the data abstractors’ through misinterpretation of chart entries or miscoding of data during data abstraction leading to either random or systematic errors. To minimise the effect of those shortcomings, recommended measures from published methodological standards^22^ have been implemented in the design of WP2.

The main strengths of the study are the very large population included in WP1, and its multifaceted, multicentre approach to understanding barriers and facilitators in performing PHECG.

## Ethics and dissemination

The study has been approved by the London-Hampstead Research Ethics Committee (ref: 18LO1679). For patient data used in WP1 and WP2 without patient consent, we have obtained section 251 support from the Confidentiality Advisory Group (ref: 18CAG0164) issued by the Health Research Authority. Participating EMS sites will conduct the study in compliance with the protocol. The study is overseen by a study management group and steering committee with an independent chair.

Findings from this study will be submitted for publication in peer-reviewed scientific journals. We will also present our findings to members of the public and professional stakeholders at local meetings in the three participating EMS, at internal academic seminars, and at national and international conferences relevant to emergency cardiovascular care.
